# Prevalence and Antimicrobial Resistance Trends of Canine Uropathogens in a Veterinary Teaching Hospital in Northern Italy: A 10-Year Retrospective Study (2014–2023)

**DOI:** 10.3390/vetsci12090910

**Published:** 2025-09-19

**Authors:** Filippo Tagliasacchi, Jari Zambarbieri, Guido Grilli, Valerio Bronzo, Emanuele Giacobbe Zampollo, Angelica Stranieri, Sara Pansecchi, Piera Anna Martino, Paola Scarpa

**Affiliations:** 1 Department of Veterinary Medicine and Animal Sciences, University of Milan, 26900 Lodi, Italy; filippo.tagliasacchi@unimi.it (F.T.); guido.grilli@unimi.it (G.G.); valerio.bronzo@unimi.it (V.B.); emanuele.zampollo@guest.unimi.it (E.G.Z.); angelica.stranieri@unimi.it (A.S.); sarapansecchi@gmail.com (S.P.); paola.scarpa@unimi.it (P.S.); 2 One Health Unit, Department of Biomedical, Surgical and Dental Sciences, University of Milan, 20133 Milan, Italy; piera.martino@unimi.it

**Keywords:** dog, urine culture, urinary tract infection, antibiotic, AMR, resistance

## Abstract

Urinary tract infections (UTIs) are a major driver of antimicrobial use in canine veterinary practice. The International Society for Companion Animal Infectious Diseases (ISCAID) guidelines recommend that empirical antimicrobial therapy be guided by locally updated data on pathogen prevalence and resistance patterns. This retrospective study analyzed 201 positive urine cultures obtained via cystocentesis from dogs at a veterinary teaching hospital in northern Italy between 2014 and 2023. The aim was to assess uropathogen distribution and antimicrobial resistance trends before and after the 2019 ISCAID guideline release. *Escherichia coli* was the predominant isolate (47.5%) showing stable prevalence over time. High resistance rates were observed for amoxicillin (62.4%), whereas resistance to trimethoprim–sulfamethoxazole was lower (33.6%). Importantly, resistance to amoxicillin–clavulanate declined significantly from 52.6% to 25.6% (*p* = 0.0002). These findings suggest amoxicillin alone may be suboptimal for empirical therapy locally, while amoxicillin–clavulanate and trimethoprim–sulfamethoxazole remain suitable first-line agents. Ongoing local surveillance is essential to support evidence-based antimicrobial stewardship.

## Simple Summary

Empirical antimicrobial treatment of bacterial urinary tract infections in dogs represents a key target for antimicrobial stewardship in veterinary medicine. International guidelines published in 2019 recommend limiting empirical antibiotic use to sporadic infection episodes, guided by updated local data on bacterial prevalence and antimicrobial resistance. This retrospective study analyzed 201 positive urine cultures collected over a 10-year period (2014–2023) at a veterinary teaching hospital in northern Italy, aiming to describe bacterial prevalence and resistance trends. *Escherichia coli* emerged as the most frequently isolated pathogen. The highest resistance rate was observed for amoxicillin, whereas lower resistance levels were found for trimethoprim–sulfamethoxazole and amoxicillin–clavulanate. Notably, resistance to amoxicillin–clavulanate significantly decreased over the study period. These findings suggest that local empirical treatment protocols could favor amoxicillin–clavulanate or trimethoprim–sulfamethoxazole over amoxicillin alone, aligning with stewardship principles. Continuous surveillance remains crucial to inform empirical choices and reduce treatment failures. Overall, this study highlights the importance of tailored antimicrobial therapy and supports responsible use of antibiotics within the One Health framework, emphasizing the need to integrate local epidemiological data into clinical decision-making.

## 1. Introduction

Bacterial urinary tract infection (UTI) is a common morbidity in dogs, affecting approximately 14% of the total canine population at least once in their lifetimes [1,2,3,4,5,6]. Diagnosing a UTI in general practice can be challenging, as nearly 49% of dogs presenting with lower urinary tract signs yield negative urine cultures, raising concerns about potential antimicrobial misuse [2]. A rigorous diagnostic approach requires the presence of compatible clinical signs, urinalysis findings, and a positive urine culture with susceptibility data from a cystocentesis-collected sample [1].

In 2019, the International Society for Companion Animal Infectious Diseases (ISCAID) issued updated guidelines for diagnosing, classifying, and treating UTIs in dogs and cats [1]. The majority of UTIs are considered sporadic bacterial infections that occur as isolated episodes without underlying disease and resolve with targeted antimicrobial therapy. To minimize treatment failure and mitigate antimicrobial resistance development, antimicrobial selection should be guided by in vitro susceptibility testing. However, pending culture and susceptibility results, empirical antimicrobial therapy may be initiated [1].

ISCAID recommendations for empirical treatment of canine UTIs advocate for the use of amoxicillin (AMX) and trimethoprim–sulfamethoxazole (TMS), both also endorsed as first-line options by the Antimicrobial Advice Ad Hoc Expert Group of the European Medicines Agency (EMA) [1,7,8]. Nonetheless, empirical antimicrobial use may select for multidrug-resistant organisms and contribute to rising local resistance rates. Empirical treatment protocols should be revised if a 10% increase in resistance occurs, relative to baseline [1,3]. To monitor emerging resistance patterns, updating data about prevalence of bacterial pathogens and resistance trends over time at the local level is recommended [1]. Indeed, bacterial prevalence and antimicrobial resistance may vary across geographical regions.

Previous studies on the prevalence of canine uropathogens and antimicrobial resistance patterns have been published for Australia, North America, New Zealand, and Europe [3,4,5,6,9,10,11,12,13,14,15]. The objectives of the present study were to determine, in a population of dogs referred to a Veterinary Teaching Hospital (VTH) in northern Italy, the prevalence of bacteria isolated from positive urine culture and possible trends in antimicrobial resistance patterns over a 10-year period (2014–2023).

## 2. Materials and Methods

### 2.1. Samples

The sample population was recruited through a review of the microbiology laboratory database of the VTH of the University of Milan. Specifically, all patients from the VTH internal medicine service who had undergone urine culture testing were initially included. To contextualize the microbiological data and identify patients with UTI, available data were cross-referenced with clinical pathology records to retrieve information on urinalysis and urinary sediment analysis when available. In addition, a retrospective review of the corresponding medical records was performed to collect information on the presence or absence of clinical signs of lower urinary tract disease (LUTD).

Patient data were organized into a spreadsheet (Excel, Microsoft Corporation, Redmond, WA, USA). Only urine samples obtained via cystocentesis, or bladder biopsies collected using sterile techniques were considered eligible for recruitment. For each positive urine culture, corresponding antibiotic susceptibility tests were always available.

In cases of patients with multiple episodes of sporadic UTIs within the study period or with recurrent UTIs, only the first episode was included, with subsequent episodes excluded. For samples testing positive for multiple pathogens, all detected pathogens were reported along with their respective resistance profiles.

### 2.2. Ethical Approval

All samples were collected from client-owned dogs presented for diagnostic evaluations at the Internal Medicine Unit of the VTH, University of Milan, as part of routine clinical work-up. Written informed consent was obtained from all owners, who agreed to the use of residual material from diagnostic procedures for research purposes. According to Article 2 (“Excluded cases from the discipline”) of Legislative Decree 26/2014, which implements Directive 2010/63/EU on the protection of animals used for scientific purposes, the study falls within veterinary clinical practices conducted for “non-experimental” purposes. As specified in the Note of 24 July 2017 issued by the Italian Ministry of Health, these activities refer to “therapeutic, curative, or diagnostic procedures performed with the owner’s informed consent on animals affected by spontaneous disease, i.e., not experimentally induced.” Therefore, no additional authorization from the Animal Welfare Body was required.

### 2.3. Analytical Procedures

All collected urine samples were sent to the internal microbiology laboratory in sterile, preservative-free syringes or containers. Samples were processed by the laboratory within 30 min of collection or stored under refrigeration and processed within 24 h of receipt. The bacteriological examination was conducted by inoculating 100 μL of urine onto two types of plates:

- Brilliance™ UTI Clarity plate (Thermo Fisher Scientific, Waltham, MA, USA; distributed by Thermo Fisher Scientific S.p.A., Rodano, MI, Italy) for preliminary colony identification based on color changes in the chromogenic medium.
- Blood agar plate (Thermo Fisher Scientific, Waltham, MA, USA; distributed by Thermo Fisher Scientific S.p.A., Rodano, MI, Italy) for colony identification using matrix-assisted laser desorption/ionization time-of-flight mass spectrometry (MALDI-TOF MS).

Both types of plates were incubated simultaneously under aerobic conditions for 18 to 24 h at 36 °C. After this first incubation, plates with no or minimal bacterial growth were re-incubated and subsequently re-examined after an additional 24 h. Once bacterial colonies were isolated and identified, antibiotic susceptibility testing was performed for each bacterial isolate using the Kirby–Bauer disk diffusion method. Bacterial colonies were suspended in a saline solution until reaching a concentration of 0.5 McFarland. The suspension was applied with a sterile swab and evenly spread over the surface of a Mueller–Hinton agar plate (Thermo Fisher Scientific, Waltham, MA, USA; distributed by Thermo Fisher Scientific S.p.A., Rodano, MI, Italy). Antibiotic disks were then placed on the plate, which was incubated at 36 °C for 24 h, after which the inhibition zones around each disk were measured. For each antimicrobial molecule, the followed reference breakpoints for “resistant” and “susceptible” were in line with EUCAST and CLSI guidelines [16,17,18,19,20]. Details on the antibiotic concentrations and the corresponding susceptibility and resistance breakpoints, as defined by EUCAST and CLSI guidelines, are reported in Appendix A (Table A1).

### 2.4. Data Analysis

The statistical analysis was conducted using SPSS 29.0 (IBM, SPSS, Armonk, NY, USA) and JMP 17 Pro (SAS Inc, Cary, NC, USA) software. For analytical purposes, the sample population was divided into two groups: patients recruited between 2014–2018 and those recruited between 2019–2023. These two five-year periods span the publication year (2019) of the ISCAID guidelines.

For the subset of subjects with positive urine cultures, descriptive analysis was performed on both the total sample and the two groups corresponding to the 2014–2018 and 2019–2023 periods. Differences in breed distribution and reproductive status between the two periods were assessed using multinomial logistic regression. Since data were not normally distributed, the Mann–Whitney U test was applied to evaluate statistically significant differences in sample age across the two periods. Pearson’s Chi-square test was used to assess significant differences in the distribution of primary uropathogens isolated across the periods.

In terms of Antimicrobial Resistance (AMR), the statistical analysis focused on first-line antibiotics recommended by ISCAID guidelines, specifically AMX and TMS, along with other antibiotics commonly used in veterinary practice, including amoxicillin–clavulanate (AMC), enrofloxacin (ENR), pradofloxacin (PRA), and marbofloxacin (MAR). To evaluate potential trends in resistance rates for the primary antibiotics across the two periods, Fisher’s exact test was applied on the results of antimicrobial susceptibility tests. The statistical analysis of the antimicrobial susceptibility testing encompassed results obtained from the entire set of positive urine cultures included in the study.

## 3. Results

### 3.1. Study Population

In all, 298 positive urine cultures were identified (January 2014–December 2023). When including samples collected only by cystocentesis and excluding those of repeat submissions (n = 94) and cultures other than bacterial pathogens (n = 3), a study pool of 201 cases was determined. From 201 positive cultures, 198 were single bacterial isolates (98.5%) and three were dual isolates (1.5%). Among the cases included in the study, 86 were recorded during the 2014–2018 period, and the remaining 115 during the 2019–2023 period.

The study population consisted of 87 males (60 intact and 27 neutered) and 114 females (36 intact and 78 neutered). The median age of dogs was 10 years (I-III IQR: 6 to 12). The population included 65 mixed-breed dogs and 136 purebred dogs. Among the purebred dogs, the most frequently encountered breeds were Labrador Retriever (n = 19), Golden Retriever (n = 13), Boxer (n = 7), Beagle (n = 6), Dachshund (n = 6), Cocker Spaniel (n = 6).

### 3.2. Prevalence of Bacterial Uropathogens

#### 3.2.1. General Population

**Figure 1:**
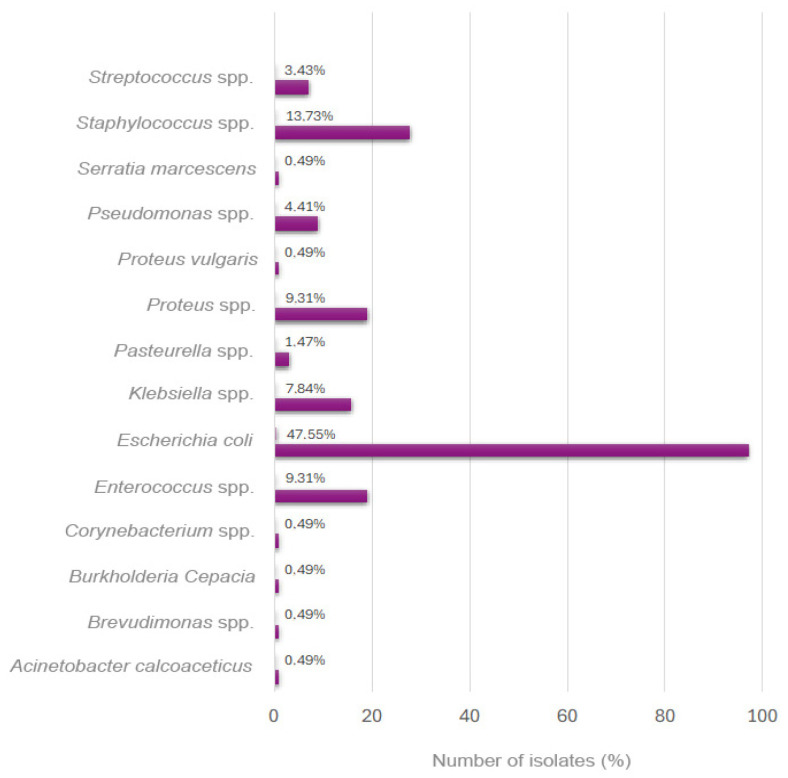
Prevalence of bacterial uropathogens isolated from positive urine cultures in the period 2014–2023. Abbreviations: spp., species.

Bacterial isolations identified *E. coli* as the predominant pathogen (n = 97; 47.5%), followed by *Staphylococcus* spp. (n = 28; 13.7%), *Enterococcus* spp. (n = 19; 9.3%), *Proteus* spp. (n = 19; 9.3%), *Klebsiella* spp. (n = 16; 7.8%), *Pseudomonas* spp. (n = 9; 4.4%), *Streptococcus* spp. (n = 7; 3.4%), *Pasteurella* spp. (n = 3; 1.4%), and six other species of lower prevalence (n = 1; 0.5%) (Figure 1).

#### 3.2.2. Comparison Between the Two Periods

When comparing the periods 2014–2018 and 2019–2023, *E coli* remained the most prevalent pathogen, with similar values in both periods (48.31% vs. 46.50%). A decreasing trend was observed for *Staphylococcus* spp. (from 19.8% to 9%) as well as for *Enterococcus* spp. (from 11.6% to 7.6%). In contrast, *Proteus* spp. showed an increase in prevalence during the 2019–2023 period (from 4.7% to 12.7%).

**Figure 2:**
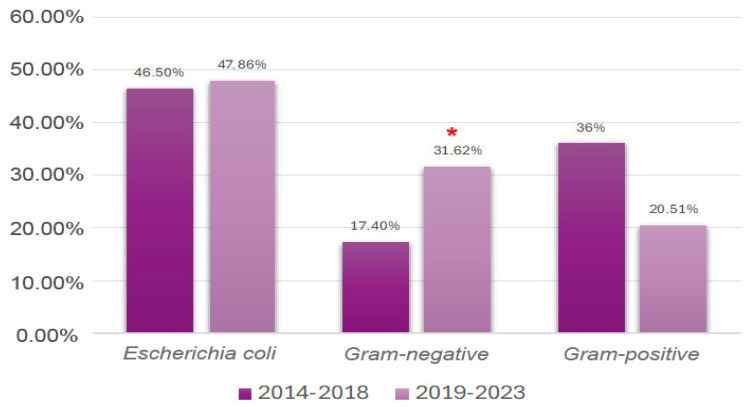
Comparison of the prevalence of uropathogens (grouped as Gram-positive, Gram-negative, and *Escherichia coli*) between the 2014–2018 and 2019–2023 periods. Legend: * indicates a statistically significant increase in Gram-negative bacteria (*p* = 0.018) between the two periods.

Overall, between the two periods, Gram-negative bacteria demonstrated a statistically significant increase (*p* = 0.018, 99%CI: 0.14 to 0.21) in prevalence, from 17.40% to 31.6% (Figure 2). The decreasing trend in percentage for Gram-positive bacteria (from 36% to 20.5%) did not reach statistical significance.

### 3.3. Trends in Antimicrobial Resistance

#### 3.3.1. First-Line Antibiotics According to ISCAID Guidelines

In the general population, AMX had an overall resistance rate of 62.4%. Trimethoprim–sulfamethoxazole showed a lower resistance rate of 33.6%.

When comparing resistance rates for these two antibiotics between the periods 2014–2018 and 2019–2023 (Table 1), no significant trends or differences emerged for AMX, which had resistance rates of 66.7% in the first period and 61.7% in the second (*p* = 0.79). For TMS, although a decreasing trend was observed (50% resistance in the 2014–2018 period and 30.4% in the 2019–2023 period), Fisher’s exact test did not indicate statistically significant differences between the two periods (*p* = 0.088).

#### 3.3.2. Other Commonly Used Antibiotics

The resistance rates observed for AMC, ENR, MAR, and PRA between 2014 and 2023 were generally lower than those recorded for first-line antibiotics. Specifically, across the entire recruited population, AMC showed an overall resistance rate of 36.4%. Similar rates were found for ENR, MAR, and PRA, with resistance rates of 36.6%, 23.5%, and 30.6%, respectively.

When comparing resistance rates between the 2014–2018 and 2019–2023 periods (Table 1), a decreasing trend was observed for AMC and PRA. Specifically, the resistance rate for AMC dropped from 52.6% in the first period to 25.6% in the second period. For PRA, the resistance rate decreased from 39.6% to 26.8%. Resistance rates for ENR and MAR remained relatively stable over time, with ENR decreasing slightly from 38.4% to 35.4% and MAR from 27% to 20.2%. Fisher’s exact test confirmed that the decrease in AMC resistance was statistically significant (*p* = 0.0002). However, statistical significance was not reached for the other antibiotics (ENR, *p* = 0.66; MAR, *p* = 0.29; PRA, *p* = 0.13).

## 4. Discussion

The present study provides an updated overview of the prevalence of bacterial uropathogens and antimicrobial resistance trends in dogs with urinary tract infections in northern Italy, allowing a regional comparison with previously published national and international data. Our findings highlight both consistencies and differences with earlier reports from Europe and other continents, particularly regarding the predominance of *E. coli* and the evolving resistance profiles of first-line and commonly used antimicrobials. By evaluating a decade-long dataset spanning the publication of the 2019 ISCAID guidelines, this study offers insights into the potential impact of antimicrobial stewardship strategies on prescribing practices and resistance patterns.

Patient demographic analysis revealed most samples came from female dogs, particularly spayed adults and seniors, with a median age of 10 years. This female predominance, especially among spayed dogs, is consistent with previous studies [6,10,21,22]. Among males, the higher prevalence of intact status aligned with findings from a 2022 study in Portugal [23].

In agreement with previous studies, *E. coli* is identified as the primary uropathogen isolated in the canine population studied, with a stable prevalence over the decade examined [12,14,15]. *E. coli* is the main bacterium also of the canine enteric flora, and the proximity between the anal and urethral sphincters promotes the development of ascending infections [24]. Additionally, virulence factors such as fimbriae, flagella, and adhesins facilitate its uroepithelial colonization [4,25,26,27]. Previous epidemiological studies on the prevalence of *E. coli* in canine UTIs have reported some variability, potentially due to differences in hospital demographics or geographical location. For instance, the overall *E. coli* prevalence observed in this study aligns with reports from other referral centers in Australia and North America [5,12,14]. However, in studies involving canine populations seen in both general and referral practices, the overall *E. coli* prevalence tended to be lower [3]. Referral centers may have a higher proportion of patients previously treated with antibiotics or affected by complicated UTIs, which could impact pathogen identification and resistance patterns. Geographical variability is also to be considered. In a recent pan-European antimicrobial surveillance program, the prevalence of *E. coli* in canine UTIs ranged from 35% to 70% [15]. Whether these differences reflect true regional variability remains uncertain. Further epidemiological studies comparing prevalence between referral institutions with that of first-opinion clinics in the territory could better frame the actual situation in northern Italy. The prevalence of *Staphylococcus* spp., *Proteus* spp., and *Enterococcus* spp. in this cohort also aligns with earlier reports [12,14,15,28,29,30].

The highest resistance rate observed in this study was for AMX, with a stable trend over the study period. Although high resistance to AMX has been widely documented in other studies [5,14,23,26,31], the resistance rate in the present population appears to be higher than previously reported. Amoxicillin is recommended by guidelines as a first-line agent for sporadic UTIs where empirical therapy is chosen. This study raises questions regarding the suitability of AMX as an empirical choice on a regional scale. The ISCAID guidelines also recommend using AMC when resistance rates for AMX are high. The low resistance rates recorded for AMC in this study, alongside the significant decreasing trend in resistance over the past decade, suggest that AMC might be a more suitable empirical choice for this region. The disparity in resistance rates between AMX and AMC is at odds with previous studies. In other populations, the two agents showed comparable resistance levels, leading authors to question the added value of clavulanic acid [1,3,32]. Although this conclusion is debated, clavulanic acid was considered as potentially exerting additional selective pressure favoring resistant pathogens. TMS, another ISCAID-recommended first-line choice for sporadic UTIs, demonstrated lower resistance rates over time. Specifically, resistance rates observed in this study for TMS are higher than in some other reports [5,14,33] but are consistent with recent studies showing resistance rates up to 51.85% [23]. These data support TMS and specifically TMS as reasonable empirical choices for canine UTIs in northern Italy, pending culture results.

Revision of empirical treatment guidelines is indicated in the face of a 10% or greater increase in AMR from baseline, in an unbiased sample population. Over the 10-year study period a statistically significant decrease in resistance rates was found to occur for AMC. This finding is consistent with what was recently reported in another Italian study that investigated antimicrobial resistance trends in uropathogenic *E. coli* isolates from a Veterinary Teaching Hospital over the same decade (2014–2023) [34]. Specifically, resistance to aminopenicillins combined with beta-lactamase inhibitors significantly decreased after 2019–2020. A significant reduction was also observed, starting between 2018 and 2020, for aminoglycosides, cephalosporins (all generations), and fluoroquinolones. In the present study, although not statistically significant, a mild downward trend in resistance rates was observed across all the antibiotics evaluated.

It is important to note that this study, similarly to the one previously mentioned, compares the years immediately before and after the publication of the ISCAID guidelines and the enforcement of European Regulation 2019/6 (implemented in Italy in 2022) [1]. These factors may have contributed to a more regulated approach to antimicrobial use in the second study period. The regulation introduced mandatory electronic veterinary prescriptions (REV), improving drug traceability, strengthening antibiotic control with penalties for non-compliance, and promoting safer, more responsible practices. These results are also consistent with recent reports showing a decline in AMR following reduced antibiotic use in Italy [35,36].

Unfortunately, no recent study has specifically addressed antimicrobial prescribing habits for the management of canine UTIs in Italy. However, a 2023 regional study conducted in Campania (southern Italy), based on the analysis of a large dataset of veterinary electronic antimicrobial prescriptions, found that enrofloxacin, amoxicillin–clavulanate, and marbofloxacin, in decreasing order, were the most frequently prescribed antimicrobials for treating canine UTIs [32].

In human medicine, medical training on antimicrobial stewardship has been proven to reduce inappropriate antibiotic use [37,38]. Future studies would be needed to evaluate veterinarians’ awareness and adherence to international guidelines in Italian clinical practice. In line with previous studies, the present data confirm that in Italy, as well as in other southern European countries (Greece, Portugal and Spain), higher resistance rates are found for all antimicrobial molecules, when compared with the northern countries (Denmark and Sweden) [15]. The lower frequency of antimicrobial resistance in northern countries is likely a consequence of the tight regulations and surveillance on antimicrobial prescribing and resistance in companion animals. In light of the present results, such strategies could be useful when aiming for the reduction in antimicrobial resistance in the Southern countries [15,31]. However, high AMR rates in this study highlight the urgent relevance of this issue locally, in line with global trends. Reports by the World Health Organization (WHO) in 2019 and 2023 emphasize prudent antibiotic use to prevent resistant infections, reduce AMR-related mortality, and curb rising healthcare costs.

Study limitations include its retrospective nature, with a non-standardized diagnostic approach and missing clinical/laboratory data, particularly from 2014–2018. Also, a retrospective study cannot reliably distinguish UTIs from subclinical bacteriuria. Throughout the decade, antimicrobial susceptibility testing was performed via disk diffusion. Minimum inhibitory concentration (MIC) testing is preferred for subtle AMR changes, though disk diffusion in this study allowed direct comparison between the two periods considered. Future studies should consider MIC testing for uropathogens. This study examined patients from a VTH internal medicine service, likely biasing the sample toward those with recurrent and/or complicated UTIs. Although providing updated prevalence and AMR data, these findings may not fully represent the clinical practice of the broader population.

## 5. Conclusions

This study describes the prevalence of canine uropathogens at a VTH in northern Italy. Consistent with other studies, *E. coli* was the most commonly identified urinary isolate. Despite a general trend towards a reduction in resistance rates, AMC was the only molecule between the evaluated antimicrobials, for which this decrease was found to be statistically significant over the study period. TMS, recommended by ISCAID as a first-line empirical antimicrobial, had a lower level of resistance compared with AMX. Waiting for the results of urine culture and antimicrobial susceptibility testing should represent, in veterinary clinical practice, the cornerstone of the therapeutic approach to canine UTIs. In the few cases where empirical therapy may be deemed necessary, these data will aid veterinarians in this region in an appropriate empirical antimicrobial choice.

## Figures and Tables

**Table 1:** Comparison of resistance rates for first-line antibiotics (amoxicillin and trimethoprim sulfamethoxazole), and other commonly used antibiotics (amoxicillin-clavulanic acid, enrofloxacin, marbofloxacin, pradofloxacin) between the study periods 2014–2018 and 2019–2023. For each antibiotic listed in the first column, the remaining columns show the percentage of resistant isolates out of the total included for each period. Specifically, from left to right, the columns indicate the resistance rate (RR%) for the entire 2014–2023 period, followed by the RR% for the 2014–2018 and 2019–2023 periods, respectively. The ‘*p* value’ column indicates whether the difference between the RR% of the two periods is statistically significant (*p* < 0.05). Abbreviations: AMC, amoxicillin–clavulanate; AMX, amoxicillin; ENR, enrofloxacin; MAR, marbofloxacin; PRA, pradofloxacin; TMS, trimethoprim–sulfamethoxazole.

Antibiotic	% Resistant 2014–2023	% Resistant 2014–2018	% Resistant 2019–2023	*p* Value
AMC	36.4%	52.6%	25.6%	**0.0002**
AMX	62.4%	66.7%	61.7%	0.79
ENR	36.6%	38.4%	35.4%	0.66
MAR	23.5%	27%	20.2%	0.29
PRA	30.6%	39.6%	26.8%	0.13
TMS	33.6%	50%	30.4%	0.088

## Data Availability

The raw data supporting the conclusions of this article will be made available by the authors on request.

## Abbreviations

The following abbreviations are used in this manuscript:

95% CI	95% confidence interval
AMC	Amoxicillin–clavulanate
AMR	Antimicrobial Resistance
AMX	Amoxicillin
EMA	European Medicines Agency
ENR	Enrofloxacin
EUCAST	European Committee on Antimicrobial Susceptibility Testing
ISCAID	International Society for Companion Animals Infectious Diseases
LUTD	Lower urinary tract disease
MALDI-TOF MS	Matrix-assisted laser desorption/ionization time-of-flight mass spectrometry
MAR	Marbofloxacin
PRA	Pradofloxacin
PU/PD	Polyuria/polydipsia
TMS	Trimethoprim–sulfamethoxazole
UTI	Urinary tract infection
VTH	Veterinary Teaching Hospital

## Appendix A

Antimicrobial susceptibility testing (AST) interpretive criteria used for bacterial isolates recovered from dogs. Table A1 summarizes the concentrations of antimicrobials in the discs (µg) and the corresponding zone diameter breakpoints (mm) applied to interpret phenotypic AST results across different bacterial taxa, including *Enterobacterales*, *Pseudomonadales*, *Staphylococcus* spp., *Enterococcus* spp., *Streptococcus* spp., and *Pasteurellaceae*. Breakpoints were derived from CLSI (VET01S, 7th Edition; M100, 33rd Edition), EUCAST (versions 15.0 and 13.0; Expected Resistant Phenotypes, version 1.2), and CASFM_VET2020 guidelines. When no official breakpoints were available, alternative criteria were applied, as specified in the table legend.

**Table A1:** Legend: ^a^ Comité de l’Antibiogramme de la Société Française de Microbiologie—CASFM—Recommandations Vétérinaires 2020; ^b^ Clinical and Laboratory Standard Istitute—CLSI VET01—Performances Standard for Antimicrobial Disk and Diluition Susceptibility Test for Bacteria Isolated From Animals, 7 th Edition. ^c^ European Committee on Suceptibility Testing—EUCAST—Expected Resistant Phenotypes, Version 1.2 January 2023; ^d^ European Committee on Suceptibility Testing–EUCAST—Breakpoint tables for interpretation of MICs and zone diameters Version 15.0; ^e^ Clinical and Laboratory Standard Istitute—CLSI- M100,—Performances Standard for Antimicrobial Susceptibility Test, 33st Ed. ^1^ The value is valid only for *Escherichia coli*. ^2^ The inhibition zones identified for Ciprofloxacin were used. ^3^ Intrinsically resistant. ^4^ Oxacillin or Cefoxitin is tested to assess susceptibility to β-lactam β-lactamase inhibitor combinations. ^5^ Susceptibility can be inferred from ampicillina. ^6^ Inhibition zones for *Staphylococcus* spp. were used. ^7^ Enrofloxacin inhibition zones were utilised. ^8^ The activity of trimethoprim–sulfamethoxazole is uncertain against enterococci, and it is not possible to predict clinical outcome. The ECOFF to categorise isolates as wild-type or non-wild-type for both *E. faecalis* and *E. faecium* is 1 mg/L, with a corresponding zone diameter ECOFF of 23 mm for trimethoprim–sulfamethoxazole.

Antimicrobial Classes	Molecule	Disk Content (µg)	Interpretive Categories and Zone Diameter Breakpoints (mm)			Source
			**S**	**I**	**R**	
			***Enterobacterales***			
Penicillins	AMX	25	≥21	20–15	<14	CASFM_VET2020 ^a^
	AMC	20/10	≥21	20–15	<14	CASFM_VET2020 ^a^
Fluoroquinolones	ENR	5	≥19	-	<19	CASFM_VET2020 ^a^
	MAR	5	≥19	-	<19	CASFM_VET2020 ^a^
	PRA	5	≥19	-	<19	CASFM_VET2020 ^a^
	PRA	5	≥24 ^1^	20–23	<19	CLSI VET01 7th Ed ^b^
Sulphonamides	TMS	1.25/23.75	≥16	15–11	<10	CASFM_VET2020 ^a^
			***Pseudomonodales***			
Penicillis	AMX	20	R ^3^	-	-	Eucast Expected Resistant Phenotype 1.2 january2023 ^c^
	AMC	20/10	R ^3^	-	-	Eucast Expected Resistant Phenotype 1.2 january2023 ^c^
Fluoroquinolones	ENR	5	22 ^2^	-	-	CASFM_VET2020 ^a^
	MAR	5	22 ^2^	-	-	CASFM_VET2020 ^a^
	PRA	5	22 ^2^	-	-	CASFM_VET2020 ^a^
Sulphonamides	TMS	1.25/23.75	R ^3^	-	-	Eucast Expected Resistant Phenotype 1.2 january2023 ^c^
			***Staphylococcus* spp.**			
Penicillins	AMX	25	20 ^4^			EUCAST 15 ^d^
	AMC	20/10	20 ^4^			EUCAST 15 ^d^
Fluoroquinolones	ENR	5	≥19		≤19	CASFM_VET2020 ^a^
	MAR	5	≥19		≤19	CASFM_VET2020 ^a^
	PRA	5	≥24	20–23	≤19	CLSI Vet 01 7th Ed ^b^
Sulphonamides	TMS	1.25/23.75	≥17		14	EUCAST v_13.1_Breakpoint_Tables ^c^
			***Enterococcus* spp.**			
Penicillins	AMX	25	≥17 ^5^			CLSI VET01 7th Ed ^b^
	AMC	20/10	≥17 ^5^			CLSI VET01 7th Ed ^b^
Fluoroquinolones	ENR	5	23 ^6^		16	CLSI VET01 7th Ed ^b^
	MAR	5	23 ^6^		16	CLSI VET01 7th Ed ^b^
	PRA	5	23 ^7^		16	CLSI VET01 7th Ed ^b^
Sulphonamides	TMS	1.25/23.75	≥23 ^8^			EUCAST v_13.1_Breakpoint_Tables. ^c^
			***Streptococcus* spp.**			
Penicillins	AMX	25	≥24 ^5^			CLSI M100 ^e^
	AMC	20/10	≥24 ^5^			CLSI M100 ^e^
Fluoroquinolones	ENR	5	≥23	17–22	≤16	CLSI Vet 01 7th Ed ^b^
	MAR	5	≥20	19–15	≤14	CLSI Vet 01 7th Ed ^b^
	PRA	5	≥23 ^7^	17–22	≤16	CLSI Vet 01 7th Ed ^b^
Sulphonamides	TMS	1.25/23.75	≥16		<10	CASFM_VET2020 ^a^
			***Pasteurellaceae***			
Penicillins	AMX	25	≥21	20–14	<14	CASFM_VET2020 ^a^
	AMC	20/10	≥21	20–14	<14	CASFM_VET2020 ^a^
Fluoroquinolones	ENR	5	≥22	21–17	<17	CASFM_VET2020 ^a^
	MAR	5	≥18	17–15	<15	CASFM_VET2020 ^a^
	PRA	5	≥24		<24	CASFM_VET2020 ^a^
Sulphonamides	TMS	1.25/23.75	≥16	15–10	<10	CASFM_VET2020 ^a^
